# Nature visits during the COVID-19 pandemic in Norway: Facilitators, motives, and associations with sociodemographic characteristics

**DOI:** 10.3389/fpubh.2023.1138915

**Published:** 2023-03-13

**Authors:** Sigbjørn Litleskare, Giovanna Calogiuri

**Affiliations:** ^1^Department of Public Health and Sport Sciences, Faculty of Social and Health Sciences, Inland Norway University of Applied Sciences, Elverum, Norway; ^2^Department of Nursing and Health Sciences, Centre for Health and Technology, University of South-Eastern Norway, Drammen, Norway

**Keywords:** nature, green exercise, green space, physical activity, lock down, nature-based activities

## Abstract

**Introduction:**

The COVID-19 pandemic has been linked to increased mental health issues and reduced well-being. Researchers also reports increased frequency of nature visits during the pandemic, proposing it may mitigate some of these negative consequences. Using the case of Norway, a country with ample access to nature and relatively low levels of pandemic-related restrictions, this study sought to (i) understand how the COVID-19 crisis impacted patterns of nature visits and specific nature-based activities, (ii) examine how these patterns varied among different population groups and levels of restrictions, and (iii) explore the motives and facilitators for increased frequency of nature visits.

**Methods:**

The data were retrieved from a national cross-sectional survey conducted in June 2021, which was designed to assess participants (*n* = 1,005, age > 15 years) habits in relation to nature visits and outdoor recreation since the beginning of the COVID-19 crisis, and associated factors.

**Results:**

The results showed that 32 % of participants increased their frequency of nature visits during the crisis, while 11 % experienced a decrease. Multivariate logistic regression revealed significant positive associations between increased frequency of nature visits and longer duration of lockdown restrictions (OR [95% CI] = 2.35 [1.28–4.29] and 4.92 [2.77–8.74] for a few weeks and several months of lockdown, respectively). Increased frequency of nature visits was also more likely among women, younger respondents, and individuals from high-income households. A Cochran's Q test showed that the most common motive for increased frequency of nature visits was “To be physically active” (74%). The most commonly reported facilitators were the possibility of using natural environments as an alternative to gyms and organized sports alongside having more time available (58 and 49%, respectively).

**Conclusion:**

These findings suggest that nature visits provided important opportunities for physical activity during the COVID-19 crisis, but also that the mental health benefits of nature visits during such times may be under-communicated. This highlights the importance of access to natural environments to promote physical activity and health, but also suggests that campaigns that specifically communicate the beneficial effects of nature visits during lockdowns or similar stressful situations might help people cope with the situation.

## 1. Introduction

The Coronavirus disease 2019 (COVID-19) pandemic has had a devastating impact on a global scale, and as of February 2023 there have been more than 750 million confirmed cases and more than 6.8 million deaths reported (1). However, the impact of COVID-19 on human health extends beyond the number of cases. Preventive measures such as minimizing contact with people outside of one's household, isolation of infected individuals, travel restrictions, and wearing masks (2) have made a significant impact on people's lives. The socio-economic impacts of the pandemic is substantial with reports of large nationwide economic losses and increased poverty rates (3). Research also reports an increase in mental health issues and a decrease of psychological wellbeing in both infected and non-infected alike (4). Some research has suggested that, because of its salutogenic effects, nature contact during the pandemic may mitigate some of the negative effects on mental health and wellbeing (5–7). Additionally, nature contact is associated with increased levels of physical activity and improved health status, both of which are considered protective factors against COVID-19 infection (8).

It has been previously proposed that the combination of nature contact and physical activity can provide synergic salutogenic benefits contributing to enhanced health (9–12). Physical activity is, by itself, a lifestyle factor that greatly influence whether people live a long and healthy life. Recent meta-analyses of studies using self-reported (13) and accelerometery-based (14) assessments of physical activity have provided conclusive evidence that greater amounts of physical activity (regardless of intensity level) are associated with a lower risk for premature mortality. An active lifestyle may also contribute to people enjoying better mental health (15, 16) and, in general, have a higher quality of life (17). Although regular physical activity is good for health by itself, in recent years a growing body of evidence has shown that physical activity in natural environments can provide health benefits above and beyond those provided by physical activity taking place in other environments (e.g., indoors or in urban settings). Natural environments such as parks and coastal areas provide attractive locations for health-enhancing physical activity (18), while on the other hand, such physical activities provide opportunities to engage with nature and enjoy the benefits provided by human-nature interactions. For instance, it was estimated that spending at least 120 minutes in nature during a regular week is associated with higher levels of self-rated health and satisfaction with life (19). Recent systematic reviews and meta-analyses reported that physical activity performed in contact with nature generally provide greater improvements to mood and wellbeing as compared to physical activity performed indoors (20) or in urban areas (21).

Research reports an increase in the frequency of human-nature interactions during the COVID-19 pandemic in different countries. As shown by a study examining longitudinal data among residents of five alpine regions, the mobility restrictions associated with the COVID-19 lockdowns generally had a negative impact on people's physical activity and exercise habits. However, this impact was less pronounced where people had the possibility to visit natural environments (22). This finding is supported by studies showing increased outdoor recreational activity during the lockdown compared to pre-lockdown periods among Strava users, with an increased use of urban green spaces, forests, and protected areas (23, 24). Several studies report that the active use of natural environments increased as a result of the pandemic and the associated restrictions (5, 23–26). This effect, however, varied between different population groups (25–28). For instance, in Norway, studies based on aggregated data from the mobile application STRAVA found a greater increase in outdoor activities among young people (24) and in areas nearby forests (23). In Australia, people with a higher socioeconomic status, who worked from home, and a reported a strong liking of natural environments, visited natural environments more frequently during the pandemic (27). A study conducted in the U.S. reported a decline in nature visits among outdoor enthusiasts (28). Furthermore, studies also highlighted how unequal access to nature was amplified during the pandemic, especially in areas with stricter mobility restrictions (6, 7).

Not only did natural environments provide opportunities for physical activity during lockdown (for those who had the possibility to visit them), research also show that nature interactions had a protective effect for mental health during lockdown periods. For example, a study of nine countries reported that those with limited nature availability in their immediate surroundings had higher odds for clinically important symptoms of depression, anxiety, and overall mental health (6). Participants also reported that contact with nature helped them to cope with these impacts, especially for those under strict lockdown (6). Other studies report similar protective effects of nature visits for mental health as well (5, 7). While these findings are in compliance with the previously mentioned salutogenic benefits of nature exposure, they also suggest that the impact of COVID-19 on human-nature interactions, and the perceived protective effect of nature contact, varies between population groups and severity of lockdown restrictions. Countries and regions with low levels of restrictions and short lockdown durations may experience less change in the frequency of nature interactions as a result of the pandemic, and the perceived protective effect against reduced mental health may be lower. Especially in a country with higher levels of weekly nature interactions compared to other countries and a generally high level of nature availability, such as Norway (29).

Norway sees high levels of participation in nature visits and outdoor recreation, a pattern supported by the fact that 61% of the population has access to safe recreational areas and natural environments (30). Moreover, outdoor recreation practice is reinforced by policies granting access to natural environments and promoting the practice within compulsory school and health institutions (31). Figures from Statistics Norway report that 97% of the population participated in some outdoor activity at least once throughout 2021 (30), and a 2016 study reported that 51% of Norwegian adults spend at least 1-h in nature-based physical activity during a regular week (29). The first confirmed Norwegian case of COVID-19 was detected on February 26th 2020. This was followed by an exponential increase in the number of cases before several lockdown measures were implemented on March 12th (32). These measures included shutdown of all school buildings, cultural and sports events, organized sports in general, people were advised, and in some case mandated to work from home and avoid leisure travel and public commute, and mandatory quarantine was implemented for people traveling to Norway from other countries, while non-residents were banned from entering the country on March 16th. Residents were also prohibited from traveling to their cabins later that month, if it was located outside their home municipalities (32). Safety measures and restrictions such as these were upheld, re-evaluated, detracted, and re-implemented to a varying degree throughout the COVID-crisis. Some measures were nationwide, while many were implemented at a municipality-level toward the later stages of the crisis. Overall, the level of restrictions in Norway has been less severe than most other western countries, as indicated by the Oxford COVID-19 Government Response Tracker (33), and visits to natural environments were allowed, and even encouraged by health institutions and outdoor recreations organizations throughout the crisis. This is in line with the relatively low infection rate; Norway was not ranked among the 60 countries with highest proportion of residents infected, according to the WHO (34).

Given the generally high accessibility to natural environments and the lively cultural atmosphere that supports and promote outdoor recreation (29), alongside the looser restrictions to outdoor physical activity during the COVID-19 crisis (33), Norway represents an interesting case for nature visits and their significance for people health and wellbeing. While some studies have investigated people's patterns of outdoor recreational activities in the Norwegian context (23, 24), these are based on aggregated data retrieved from online sources (STRAVA and Google mobility trends), which may not be representative of the entire population and did not provide insights on the participants' subjective experience of nature, as well as the motives and facilitators that supported nature-based recreation throughout the COVID-19 pandemic. Moreover, the long-term effects of lockdown are also largely unexplored, as most studies within this field were conducted in 2020 (6, 7, 23–28, 35). Hence, the purpose of this study was to investigate to what extent and how the COVID-19 crisis influenced the patterns and perceptions of nature visits among Norwegians. More specifically, the following research questions were outlined:

RQ1: To what extent did adult Norwegian increase their participation in nature visits during the COVID-19 pandemic?
H1: It was hypothesized that a large portion of Norwegians will report a relative increase in their frequency of nature visits during the COVID-19 pandemic.
RQ2: What is the association of increased participation in nature visits with local restrictions and sociodemographic characteristics?
H2: It was hypothesized that, when controlling for the participants sociodemographic characteristics the individuals who were subjected to pandemic-related restriction for a longer period will be more likely to report a relative increase in the frequency of nature visits. Moreover, associations will be observed with the participants sociodemographic characteristics, in particular, the likelihood to report increased nature visits will be associated with higher income and educational level.
RQ3: What motives and facilitators that supported increased frequency of nature visits among adult Norwegians during the COVID-19 pandemic?
H3: It was hypothesized that the increased frequency of nature visits is primarily supported by greater availability of time or flexibility in one's days. Primary motives will relate to the need of experience nature as a way to cope with pandemic situation, but also as a way keep physically active.

## 2. Materials and methods

### 2.1. Study design and sample

The data were retrieved from a national survey initiated by Norsk Friluftliv, an umbrella organization for outdoor recreations in Norway, and conducted by a professional statistical agency (Ipsos MMI)–both provided permission to use the data for research purposes. The purpose of the survey was to map, in a sample representative of the Norwegian adult population, the extent to which people perceived to have changed their habits relative to participation in nature visits and outdoor recreation, as well as possible factors associated with it, since the beginning of the COVID-19 crisis. Data was collected *via* an online survey between June 2nd and 21st 2021. The survey was distributed, using stratified sampling, to a web-panel of people who reside in Norway. The web-panel consist of approximately 97,000 people above the age of 15. A sub-sample of this web-panel was invited to participate based on age, gender, and geography, with the aim of generating a sample representative of the Norwegian population. The final sample included in this study comprised 1,005 participants.

### 2.2. Study variables

#### 2.2.1. Frequency of nature visits and specific nature-based activities

Relative frequency of nature visits–Participants answered a single question enquiring “Think of how often went for a nature walk or engaged in outdoor recreation before the pandemic (March 2020). During the pandemic, did you do these activities more, as, or less frequently?” The item also specified that if one had never done these activities before, but initiate them during the pandemic, they should select the response option “more often”. A response option “I never engaged in these activities” was also provided.

Specific nature-based activities—The participants were also presented with a list of specific nature-based activities (went for walk, having a meal outdoors, camping, fishing, used a canoe, climbing, organized outdoors activities etc.), and were asked to indicated how often (“For the first time”; “More frequently”; “As frequently”; “Less frequently”; and “I never engaged in this activity”) they engaged in each activity during the pandemic. A reference time to indicate the beginning of the pandemic period was provided as “before March 2020”.

#### 2.2.2. Sociodemographic characteristics and severity of restrictions

Sociodemographic characteristics—Participants were asked about relevant demographic information, including age (years), gender (male, female), centrality (whether one lives in a city, a town, a village, or a rural area), income (response options: “>300,000 NOK”; “300,000−499,999 NOK”; “500,00−799,999 NOK”; “800,000−999,999 NOK”; “1,000,000−1,499,999 NOK”; “≥1,500,000 NOK”; “I don't know/don't want to report”), and highest completed educational level (response option: “Primary school”; “Upper-level school”, “Bachelor degree”; “Master's degree or higher”). All sociodemographic variables were self-reported.

Duration of restrictions—This was assessed through an item inquiring “The corona situation has affected different parts of the country differently. Approximately, how many months in the past year did the “lockdown” last where you live?” The caption also provided a definition of lockdown as “The closure of workplaces, restaurants, cultural facilities, shops etc.” The respondents were asked to select the response option that best fit the among the following: “Several months”, “some weeks”, “Almost nothing”, and “I don't know”.

#### 2.2.3. Facilitators and motives for participation in nature visits

These measurements were provided only for participants that reported to have increased their frequency of nature visits during the pandemic. Multiple answers were possible in relation to both motives and facilitators.

Facilitators for increased nature visits—Participants were presented with a list of five statements outlining facilitators relative to nature and nature-based activities during the pandemic (e.g., “I had more time available” and “Friends and family had more time to accompany me outdoors”). All items were assessed at a binary level, with the respondents being asked to select all motives that applied to them. In the caption, “March 2020” was again provided as time reference to indicate the beginning of the pandemic.

Motives for increased nature visits—Participants were presented with a list of five statements relative to motives for and reasons that allowed higher frequency of nature visits during the pandemic (e.g., “Nature helped me to cope with the pandemic” and “I used nature to be physically active”). A filter was applied, so that only the respondents who reported to have visited nature more frequently would respond to these items. All items were assessed at a binary level, with the respondents being asked to select all motives that applied to them. In the caption, “March 2020” was provided as time reference to indicate the beginning of the pandemic.

### 2.3. Analyses

To address RQ1, relative frequency of nature visits (*n* = 1,004) and specific nature-based activities (*n* = 1,005 were examined through descriptive statistics and presented as frequencies (*n*) and percentages (%).

To assess the associations between sociodemographic characteristics and reported changes in the frequency of nature visits (RQ2), multivariate binomial logistic regression was used to calculate odds-ratios (OR) with 95 % confidence intervals (CI) for the participants' sociodemographic characteristics and experienced time in lockdown (*n* = 1,004). The variable “frequency of nature visits” was dichotomized for the purpose of this analysis, comparing those who increased the frequency vs. those who did not. Gender, age, household income, education, total duration of restrictions, and centrality were entered as independent variables in the analysis following assessment of independence of observations. Age was categorized as 15–34, 35–54, and 55+ (15–34 used as reference), household income was categorized as 499,999 NOK or less, 500,000–999,999 NOK, and 1 000,000 NOK or more (1 000,000 NOK or more used as reference). For education, the two lowest levels of education were combined to one category. For gender, men was used as the reference category, and for age and education the lowest category was used as reference, for centrality the response “City” was used as reference, and for lockdown “Almost non-existent” was used as reference. Participants that answered “don't know” for any of these questions were excluded from the analysis for that particular question.

Finally, to address RQ3, Cochrans Q with Bonferroni adjustment was used to assess potential significant differences in the frequency distribution of facilitators and motives for increased frequency of nature visits. The analysis was performed on the subset of participants who reported to have increased the frequency of their nature visits since the announcement of the first lockdown (*n* = 324). This provided an assessment of which facilitators and motives were most commonly reported by the participants who reported to have increased their nature visits frequency.

Data were checked for distribution, missing values, and outliers prior to the analysis. Examinations were also conducted to assure that relevant assumptions for the specific statistical tests were met. All statistical analyses were performed in Statistical Package for the Social Sciences (SPSS) version 28 (IBM Corporation, Armonk, NY, USA). The significance level was set at *p* < 0.05.

## 3. Results

The sample was fairly balanced in terms of sociodemographic characteristics (Table 1). The majority of participants resided in cities (35.4 %) and experienced several months of lockdown (56.6 %). Only 5.9% of participants reported that they missed having better access to nature in their neighborhood and only 3.9% experienced unemployment or forced leave after the start of lockdown –because of the small frequencies, these variables were not included in the final multivariate analyses (to be noted that binary logistic regressions showed no association of perceived access to nature with increase nature visits, while a significant and positive association was found for experiencing a challenging job situation and increased nature visits [OR = 2.29, 95% C.I. = 1.20–4.35, *p* = 0.012]).

**Table 1:** Sociodemographic characteristics and duration of lockdown restrictions for all participants (*n* = 1,005).

**Variable**	***n* (%)**
**Gender**	
Male	485 (48.3)
Female	520 (51.7)
**Age (years)**	
15–34	254 (25.3)
35–54	353 (35.1)
≥ 55	398 (39.6)
**Education**	
Upper-level school or lower	361 (35.9)
Bachelor degree	373 (37.1)
Master's degree or higher	271 (27.0)
**Income household (NOK)**	
1,000,000 -	354 (35.2)
500,000–999,999	385 (38.3)
0–499,999	161 (16.0)
Don't know/don't want to report	105 (10.4)
**Centrality**	
Larger city	356 (35.4)
Smaller city/town	266 (26.5)
Village	222 (22.1)
Rural area	161 (16.0)
**Lockdown duration**	
Almost non-existent	153 (15.2)
A few weeks	256 (25.5)
Several months	569 (56.6)
Don't know	25 (2.5)

The relative frequency of nature visits and specific nature-based activities during the pandemic are reported in Table 2. Most participants maintained their usual level of nature visits (56.8%, including those who reported to have never visited nature), but the number of people who increased the frequency of nature visits was larger (32.3%) compared to the those who decreased it (10.8%), leading to a net increase of nature visits during the pandemic. The specific nature-based activities that increased the most were walks in nature (39.0%), eating a meal outdoors (23.4%), and skiing (15.6%; Table 2). On the other hand, organized outdoor activities, climbing, sailing or using a rowboat, hunting, and fishing experienced a net decrease during the pandemic.

**Table 2:** Proportion of participants who either increased, maintained, reduced, or never engage in nature-based activities (*n* = 1,005).

**Nature visits**	**More often than before**	**Same as before**	**Less than before**	**Never do this**
	***n*** **(%)**	***n*** **(%)**	***n*** **(%)**	***n*** **(%)**
Frequency of nature visits	324 (32.29)	559 (55.6)	109 (10.8)	12 (1.2)
**Specific activities**				
Go for a walk	392 (39.0)	528 (52.5)	67 (6.7)	18 (1.8)
Eating a meal	235 (23.4)	561 (55.8)	80 (8.0)	129 (12.8)
Skiing	152 (15.6)	432 (43.0)	127 (12.69)	294 (29.3)
Lighting a fire	151 (15.0)	419 (41.7)	90 (9.0)	345 (34.3)
Biking	146 (14.5)	470 (46.8)	126 (12.5)	263 (26.2)
Swimming	108 (10.7)	619 (61.6)	105 (10.4)	173 (17.2)
Spending the night	100 (10.0)	279 (27.8)	71 (7.1)	555 (55.2)
Picking berries	85 (8.5)	513 (51.0)	76 (7.6)	331 (32.9)
Orienteering	68 (6.8)	120 (11.9)	38 (3.8)	779 (77.5)
Kayaking or canoeing	66 (6.6)	198 (19.7)	57 (5.7)	684 (68.1)
Fishing	63 (6.3)	347 (34.5)	104 (10.3)	491 (48.9)
Picking mushrooms	56 (5.6)	296 (29.5)	41 (4.1)	612 (60.9)
Organized activities	38 (3.8)	166 (16.5)	110 (10.9)	691 (68.8)
Climbing	34 (3.4)	117 (11.6)	53 (5.3)	801 (79.7)
Sailing or rowboat	22 (2.2)	236 (23.5)	61 (6.1)	686 (68.3)
Hunting	21 (2.1)	111 (11.0)	33 (3.3)	840 (83.6)

The results of the logistic regression analysis revealed that women, younger people, and respondents with higher household income were more likely to report increased frequency of nature visits compared to men, older age groups, and people in the middle-income category (Table 3). A significant association also emerged for the duration of the lockdown, with those who reported being affected by pandemic-related restrictions for “a few weeks” or “several months” being more likely to report increased nature visits compared with those who reported to have experienced “almost non-existing” restrictions. No significant associations were found between increased frequency of nature visits and educational level or centrality.

**Table 3:** Sociodemographic characteristics and lockdown duration, for participants that increased the frequency of nature visits and those who did not, and their associations with increased frequency of nature visits reported as odds ratios with 95% confidence interval (*n* = 1,004).

**Variable**	**↑frequency *n* (%)**	**↓frequency *n* (%)**	**Odds ratio**	**95% CI**	**p**
**Gender**					
Male	125 (38.6)	360 (52.9)			
Female	199 (61.4)	320 (47.1)	1.83	1.35–2.47	< 0.001
**Age (years)**					
15–34	101 (31.2)	153 (22.5)			
35–54	110 (34.0)	242 (35.6)	0.64	0.43–0.96	0.030
≥ 55	113 (34.9)	285 (41.9)	0.55	0.37–0.82	0.004
**Education**					
Upper-level school or lower	112 (34.6)	249 (36.6)			
Bachelor degree	112 (34.6)	260 (38.2)	0.92	0.63–1.34	0.675
Master's degree or higher	100 (30.9)	171 (25.1)	1.18	0.79–1.77	0.424
**Income household (NOK)**					
1,000,000 -	135 (47.0)	219 (35.8)			
500,000–999,999	102 (35.5)	283 (46.2)	0.63	0.46–0.89	0.007
0–499,999	50 (17.4)	110 (18.0)	0.76	0.49–1.19	0.229
**Centrality**					
Larger city	131 (40.4)	224 (32.9)			
Smaller city/town	84 (25.9)	182 (26.8)	0.80	0.49–1.31	0.374
Village	65 (20.1)	157 (23.1)	0.81	0.49–1.34	0.407
Rural area	44 (13.6)	117 (17.2)	0.81	0.48–1.36	0.423
**Lockdown duration**					
Almost non-existent	24 (7.5)	129 (19.6)			
A few weeks	66 (20.8)	190 (28.8)	2.35	1.28–4.29	0.006
Several months	228 (71.7)	340 (51.6)	4.92	2.77–8.74	< 0.001

↑frequency = increased frequency of nature visits. ↓frequency = same or decreased frequency of nature visits. n, number of participants; CI, confidence interval.

“More time available” and nature visits as an “Alternative to gyms and organized sports” were the most commonly reported facilitators for increased nature visits. Both of these facilitators were found to have a higher frequency than the three other facilitators [χ^2^(4) = 174.647, *p* < 0.01]. “More flexible days” was also reported by significantly more people compared to “Shorter distance to nature due to change in work/school situation” (Table 4; *p* < 0.01). “To be physically active” than the four other motives [χ^2^(4) = 122.541, p < 0.01]. There were no other significant differences between any of the facilitators or the motives (Table 4).

**Table 4:** Facilitators and motives for increased frequency among participants that increased their frequency of nature visits (*n* = 324).

**Facilitators**	***n***
Alternative to gyms and organized sports	189
More time available	159
More flexible days	90^a, b^
Friends and family had more time to accompany me	62^a, b^
Shorter distance to nature due to change in work/school situation	34^a, b, c^
**Motives**	
To be physically active	241
Meeting friends and family	157^1^
To reduce stress and worries	139^1^
To cope with the pandemic situation	137^1^
To experience quietness and calm	124^1^

^a^Significantly different from “More time available”, ^b^Significantly different from “Alternative to gyms and organized sports”, ^c^Significantly different from “More flexible days”. ^1^Significantly different from “To be physically active”. P < 0.05.

## 4. Discussion

The results indicate that, while most adults in Norway reported no change in nature visits during the COVID-19 crisis, about one third reported an increased frequency. In particular, walks in nature, eating a meal outdoors, and skiing were the activities in which most people reported to have engaged in more frequently. The increased frequency of nature visits was larger among those who experienced longer duration of lockdown-related restrictions, compared with those who experience shorter or no restrictions. Moreover, increased frequency of nature visits was more prevalent among women, younger respondents (15–34 years), and people in the highest household income category, compared to men, older age groups, and people in the middle-income category, respectively. The two most commonly reported facilitators for increased frequency of nature visits were seeing nature visits as a replacement for gym- of sports activities and having more time available. The most commonly reported motive for nature visits during the pandemic was physical activity.

These findings are in line with previous research (5, 23–26), and show that even countries with relatively few pandemic-related mobility restrictions, and with high baseline level of nature interactions, experienced an increase in nature visits as a result of the crisis. Notably, the total duration of lockdown in the region emerged as the main driving factor for the increased frequency of nature visits, at least among the factors included in this study, with an odds-ratio of 4.92 for participants experiencing several months of lockdown compared with those reporting almost non-existing restrictions. Furthermore, the findings indicate that the increase in nature visits was primarily motivated by finding alternative opportunities for exercising and engaging in physical activity, as during lockdown gyms and organized sports were shut down. This forced change, was also facilitated by having more time available. Moreover, it must be considered that this was likely possible thanks to specific policies in Norway that protect and facilitate access to and utilization of natural environments for physical activities and recreation. This relates to general national policies such as the “The Outdoor Recreation Act” [a law that codifies the people's right to access and exploit the wilderness for recreations purposes (36)] as well as investments made by many municipalities in maintaining physical activity-supportive features such as lighted paths in forests and cross-country skiing trails. Importantly, in Norway, no restrictions were set on access of parks and other natural environments. All of this is in line with a recent analysis by Schöttl et al. (22), which indicated that the impact of the lockdown measures on people's physical activity levels tended to be less pronounced where people had the possibility to visit natural environments. These findings highlight the paramount importance of natural environments as an arena for the promotion of health-enhancing physical activity in the population, and corroborate the value of investing in policies that grant and facilitate the access to them.

While a higher likelihood of increased nature visits was found for respondents in the highest household income category, no association between educational level and change in frequency of nature visits in the present study were found, which contradicts previous research. For instance, Australian (27) and Swiss research (26) reported that higher educational level was associated with increased access to natural environments during the pandemic. Additionally, educational level is a known determinant of healthy behaviors, including physical activity, in the Norwegian context (37). This may be explained by differences in how these variables were measured, but they may also indicate important differences between countries. Evidence exists indicating that, in Norway, participation in nature-based physical activity may be less subjected to social gradients compared with other forms of physical activity. This is highlighted by a smaller difference in weekly nature visits across socio-economic strata, although a social gradient was found for the extent to which people with different educational level and household income saw limited access to nature as a barrier to increase their nature visits (29). In the present study, however, only 5.9 % of participants in the present study complaint about limited access to nature, which was not associated with the respondents' relative frequency of nature visits during the pandemic (although this variable could not be included in the multivariate model because of its highly skewed distribution). Hence, some social inequalities may exist in the extent to which natural environments may be accessible to different social groups in Norway, but the high level of nature availability in the population might reduce the impact of socioeconomic differences in frequency of nature visits, resulting in a different pattern of associations between socioeconomic factors and the change in frequency of nature visits during a pandemic when compared to other countries.

The findings indicate that the increased frequency of nature visits during the pandemic occurred to a greater extent among women, younger people, and individuals with higher household income is in accordance with previous research from Belgium (25), Norway (24), and Australia (27). As women, younger adults, and people with high household income typically tend to exercise more in gyms and sport facilities (29), in line with the finding on physical activity motivation, this suggests that the increased frequency of nature visits, at least in the Norwegian context, may be primarily seen as an exercise-compensation phenomenon. This would imply that the increased nature visits were primarily the result of people shifting their exercise setting away from gyms or sport facilities to natural environment, rather than a general increased need for nature contact during the COVID-19 crisis. Even though previous research has demonstrated benefits to health and well-being of nature visits and green exercise (21, 38), also in the context of the COVID-19 pandemic (5–7), using nature as a means to cope with the pandemic situation was less frequently reported as a motive in this study. On the one hand, this may be read in light of the generally high levels of nature visits in the Norwegian population (29), which is supported by a general social discourse that recognize health and wellbeing benefits to nature experience (39). Since many individuals already use nature as a means to gain wellbeing benefits, they may be less likely to see this as a primary motive driving their increased frequency of nature visits during the COVID-19 pandemic. On the other hand, this may be seen, again, in light of the exercise-compensation interpretation. Gaining wellbeing benefits and reduce stress are important motives supporting exercise and physical activity, gym-, sport- and nature-based exercise alike (40). Since the increase in nature visits was primarily driven by exercise and physical activity purposes, those who increased their nature visits may see these benefits as part of the exercise and physical activity experience, rather than as a distinct coping strategy. Nevertheless, these individuals may have “unintentionally” gained the additional benefits provided by nature exposure. On the other hand, the fact that not more people reported to engage in nature visits specifically to cope with the pandemic may also be negatively connotated, as in times like these people could benefit greatly from nature visits due to its positive effects on stress, depression, anxiety, and overall wellbeing (21, 38), which show increased prevalence during the crisis (4). This suggests that campaigns that specifically communicate the beneficial effects of nature visits during lockdowns or similar stressful situations might help people to cope with the situation. This is particularly meaningful for the ~10 % of participants in this study that reported reduced frequency of nature visits during the pandemic. One simple and accessible activity is going for a walk in a natural environment. In fact, this study found that only 1.8% of participants never go out for a nature walk, making it the most popular nature activity among participants, which aligns with findings from other national surveys (41). During the pandemic, nature walks were also the activity that saw the greatest increase in participation. Previous research has shown that nature walks can provide numerous benefits to health and wellbeing, such as reduced stress, anxiety, and depression, as well as improved mood and affect (42–44).

### 4.1. Limitations

The COVID-19 crisis had a major impact on society and led to rapid changes in population trends and behaviors as lockdown measures were implemented and revoked. In these uncertain times, the cross-sectional design allows researchers to act quickly to capture a snapshot of the impact of these changes. However, a cross-sectional design, based solely on self-reported measurements, does not allow for the investigation of causal relationships between variables or strict control of confounders. Additionally, the use of self-reported data can be prone to bias, as participants may not accurately report their experiences. Future research should aim to understand whether the increase in nature visits during the pandemic leads to long-term changes to people's nature interactions or if it is a transient increase that returns to normal when restrictions are no longer in effect. Potential positive effects of nature visits on the negative health impact of long-COVID would also be an interesting avenue to pursue.

### 4.2. Conclusion

Overall, nature visits increased among adult Norwegians since the start of the COVID-19 crisis (March 2020). This increase was clearly associated with the duration of pandemic-related restrictions and appear to be primarily driven by the need of finding alternative opportunities for engaging in physical activity during the lockdown periods. These findings confirm the public health significance of granting and promoting access to nature for physical activity and wellbeing purposes, even in a country with less stringent restrictions compared with other countries, and that generally show high baseline level of nature-based recreation. At the same time, the findings also suggest that the mental health and wellbeing benefits of nature visits during periods of high psychological strains (such as the COVID-19 crisis) may be under-communicated.

## Data availability statement

The raw data supporting the conclusions of this article will be made available by the authors, without undue reservation.

## Ethics statement

Ethical review and approval was not required for the study on human participants in accordance with the local legislation and institutional requirements. Written informed consent from the participants' legal guardian/next of kin was not required to participate in this study in accordance with the national legislation and the institutional requirements.

## Author contributions

GC was the primary person responsible for the conception of the study and made substantial contributions to the manuscript. SL participated in the conception of the study, drafted the manuscript, and carried out the analysis in collaboration with GC. SL and GC approved the final version. Both authors contributed to the article and approved the submitted version.
